# Chronic pain in female breast cancer survivors - prevalence, characteristics and contributing factors: a cross-sectional pilot study

**DOI:** 10.1186/s12905-023-02766-6

**Published:** 2023-11-16

**Authors:** Nelisiwe Shabangu, Tselane Thebe, Michelle Casey, Ursula Wesselmann, Romy Parker

**Affiliations:** 1 Department of Anaesthesia and Perioperative Medicine, Faculty of Health Sciences, University of Cape Town and Groote Schuur Hospital. Cape Town, Cape Town, South Africa; 2 Department of Oncology, Stellenbosch University and Tygerberg Hospital, Cape Town, South Africa; 3 Department of Anesthesiology and Perioperative Medicine, Division of Pain Medicine, Department of Neurology, Department of Psychology, The University of Alabama at Birmingham, Birmingham, AL 35294 USA

**Keywords:** Breast cancer survivor, Chronic pain, Prevalence

## Abstract

**Background:**

While the global incidence of breast cancer is increasing, there is also an increase in the numbers of breast cancer survivors and in survival duration, as early detection programs are implemented, and treatments are optimized. Breast cancer survivors in several countries commonly struggle with a range of symptoms (fatigue, insomnia, depression) with 25–80% of survivors suffering from chronic pain. There is a paucity of literature reporting on breast cancer survivors in South Africa. In this pilot study we aimed to determine the prevalence of chronic pain in female breast cancer survivors attending the breast oncology clinic.

**Methods:**

A cross-sectional survey was conducted of all breast cancer survivors attending the Groote Schuur Hospital Breast Unit during one month in 2019. 44 female breast cancer survivors (median age 60.5y) completed a sociodemographic questionnaire, the Brief Pain Inventory, Pain Catastrophizing Scale and measures for neuropathic pain (DN4), health related quality of life (HRQoL; EQ-5d-3 L), physical activity (IPAQ), depression and anxiety (PHQ4), and screening questions to evaluate sleep, happiness and perceived discrimination in the language of their choice.

**Results:**

The prevalence of chronic pain (pain on most days for more than three months) was 59% (95%CI 44–72), a significantly higher number than the 18,3% prevalence of chronic pain reported by South African adults. 39% of the women were classified as having neuropathic pain. The median pain severity score was 3.75 (IQR = 2.75-5) and the median pain interference with function score was 4 (IQR = 2.9–5.4). The women were experiencing pain in a median of 2 different body sites (IQR = 1–3). The women with pain were more likely to be unemployed or receiving a disability grant, had significantly worse HRQoL, and significantly worse scores for risk of depression and anxiety.

**Conclusion:**

The results of this pilot study suggest that chronic pain may be a significant burden for South African breast cancer survivors. Routine screening for chronic pain in breast cancer survivors is recommended with a larger study indicated to explore this issue further.

**Supplementary Information:**

The online version contains supplementary material available at 10.1186/s12905-023-02766-6.

## Background

Globally, more women die of breast cancer than any other type of cancer [1]. Approximately 600 000 women died of breast cancer in 2020 with the majority of these women living in sub-Saharan Africa [2]. The incidence of breast cancer is increasing, and it is estimated that over 19 million women will suffer from breast cancer by 2025 [3]. The increasing incidence of breast cancer is associated with increased survival as early detection programs are implemented and treatments are optimized [1]. Breast cancer survival rates are 86% in the USA and although presently only at 40% in sub-Saharan Africa, survival rates are improving. A major new collaborative effort, the Global Breast Cancer Initiative, was introduced in recognition of International Women’s Day in March 2021 by the World Health Organization, with the objective of reducing global breast cancer mortality by 2.5% per year until 2040, thereby averting an estimated 2.5 million deaths [4]. Therefore, while there will be a growing number of women developing breast cancer, there will also be a growing number of women surviving breast cancer.

Breast cancer survivors commonly struggle with ongoing symptoms such as fatigue, insomnia, depression and chronic pain [5]. Pain and pain-related disability affect 25–80% of the survivorship population [5]. The prevalence of chronic pain appears to be similar in the limited existing data from South Africa. The neuropathic condition, Post Mastectomy Pain Syndrome, had a 38% prevalence in 92 women who had received treatment at Chris Hani Baragwanath Hospital in South Africa [6]. Literature reporting on the quality of life of African Breast Cancer survivors reports chronic pain as a common symptom negatively impacting quality of life in a wide variety of populations (Nigeria, South Africa and Morocco) [7].

Most concerningly, persistent pain has been reported to be associated with shorter survival time [8] and severe chronic pain is associated with increased risk of mortality, independent of socio-demographic factors [9]. Living with chronic pain (pain that has been present on most days for longer than three months) negatively impacts on quality of life, places greater financial demand on the individual and on the health system and may result in reduced activities and restricted participation in meaningful life roles [10]. Treatment of chronic pain in breast cancer survivors is indicated, not only to decrease the suffering of the individual, but to optimize quality of life and restore patients to their pre-morbid function and their participation in meaningful life roles such as in family, social networks and work.

Numerous biopsychosocial variables have been identified as increasing the risk of developing chronic pain, both in breast cancer survivors and in other populations. Known risk factors for developing chronic pain include: being female; having a lower level of education; lower socioeconomic status; being unemployed; economic insecurity; having low levels of social support; smoking, low levels of physical activity, poor sleep quality, depression, anxiety, and pain catastrophizing [11–13]. In breast cancer survivors specifically, different cancer treatments also have different chronic pain risk profiles [14]. In addition, some studies have shown that younger age at diagnosis of breast cancer is associated with higher prevalence of chronic pain [15]. The majority of these studies have been conducted in breast cancer survivors living in the developed world.

There is a paucity of evidence regarding chronic pain in breast cancer survivors from South Africa. This is concerning as recent literature indicates that the prevalence of breast cancer in sub-Saharan Africa will double by 2050. Describing the prevalence, characteristics and contributing factors to chronic pain in South African breast cancer survivors, will inform strategies to reduce and manage the condition.

The aim of this pilot study was to determine the prevalence of chronic pain in female breast cancer survivors attending the Groote Schuur Hospital (GSH) breast oncology clinic (Breast Unit) during one month in 2019. In addition, the socio-demographic and clinical characteristics and potential contributing factors were explored. These data will be used to guide a larger cross-sectional study to determine the prevalence of chronic pain in female breast cancer survivors in South Africa.

## Methods

A cross-sectional cohort descriptive pilot study was conducted during one month in 2019. All female breast cancer survivors being followed up at the GSH Breast Unit during the data collection period were invited to participate in the study. Inclusion criteria included: being female, breast cancer survivor (completed active treatment and in remission i.e. for luminal breast cancer survivors a minimum of four months since completing treatment, for non-luminal, a minimum of six months since completing treatment), and able to speak and understand either English, Afrikaans or isiXhosa. Different time periods were selected for survivors of luminal vs. non-luminal cancer based on GSH Breast Unit policy of following up luminal cancer survivors earlier. Patients with cognitive impairment or intellectual disability were excluded from the study.

For the purposes of this study, a breast cancer survivor was defined as a patient who has completed their active treatment with curative intent and is now being monitored. The sampling frame was female breast cancer survivors who had completed treatment at the GSH Breast Unit and had attended for follow up during one calendar month in 2019. During 2017 and 2018, the Breast Unit saw an average of 51 new, and 380 follow up patients per month. Based on these data, we estimated that a maximum of 51 breast cancer survivors attend the Breast Unit per month. The sample size required for a cross-sectional survey was calculated using the Yamane formula [16]. The goal was to recruit a minimum of 45 patients for the results to be generalizable to the sampling frame.

## Measurement instruments

A sociodemographic questionnaire was developed to capture information previously described as increasing the risk of developing chronic pain including age, marital status, level of education, employment status, economic profile and health literacy. Health literacy was established using the SOS Mnemonic [17]. The IMMPACT group recommend that pain be evaluated using instruments that capture both the complexity and variability of the pain experience as well as the effect of pain on the individual’s activities and participation. As it fulfills these criteria, and has been translated and validated for numerous populations, including in South African English, Afrikaans and isiXhosa, the Brief Pain Inventory (BPI) was selected [18]. The BPI generates a Pain Severity Score (PSS), a Pain Interference Score (PIS) and a Pain Management Index (PMI). In addition, the BPI allows participants to indicate different sites of pain on a body chart, facilitating the identification of multiple sites of pain which are an indicator of chronic nociplastic pain [19].

The BPI uses an initial screening question which allows for the identification of chronic pain. The question normalizes daily pain experiences and highlights to patients the difference between chronic pain and “normal daily pains”. The question states: “Throughout our lives most of us have had pain from time to time (such as minor headaches, sprains and toothaches). Have you had pain other than these everyday kinds of pain”. To establish the presence of chronic pain the question was modified to state “Have you had pain other than these everyday kinds of pain on ***most days during the last three months***?” Participants who responded “Yes” to this question were classified as having chronic pain and completed the full BPI, participants responding “No” were classified as not having chronic pain and did not complete the BPI but did complete all other instruments.

To screen for neuropathic pain we used the Douleur Neuropathique en 4 questionnaire (DN4), a validated clinician-administered questionnaire [20]. The DN4 had been found to be a reliable tool to screen for the presence of neuropathic pain in relevant anatomical areas following chemotherapy administered for the treatment of breast cancer and following surgery for the resection of breast tumours.

The EQ-5D-3 L was used to evaluate health-related quality of life in terms of general health-status and health profile [21]. This assessment tool utilizes five categories: mobility, self-care, usual activities, pain/discomfort and anxiety/depression. The EQ-5D-3 L instrument had been translated and validated in isiXhosa [21] and Afrikaans [22]. Furthermore, it had been used in several studies to assess the quality of life in breast cancer survivors.

We used the International Physical Activity Questionnaire (IPAQ) to assess self-reported physical activity [23]. The Short Version consists of seven core questions, which assesses the respondent’s Perceived Physical Activity during the “last seven days” (prior week to completing the questionnaire).

Depression and anxiety have both been consistently associated with chronic pain with some authors suggesting that 50% of patients with a chronic pain condition will also present with a depressive disorder [24]. We used the four-item Patient Health Questionnaire (PHQ-4) to screen for both anxiety and depression [25]. The PHQ-4 includes two questions on depression PHQ-2, and two questions on anxiety.

We used the South African version of the Pain Catastrophizing Scale (PCS) to measure pain catastrophizing [26]. The PCS is available in South African English, Afrikaans and isiXhosa and has been validated for use in English and Afrikaans South African populations. To reduce questionnaire burden, quick screening questions to evaluate sleep, happiness and perceived discrimination were used. A single question from the PHQ-9 was used to screen for the presence or absence of sleep disturbance [27]. A single question to assess Happiness was used based on the work of Calvo [28]: “If you were to consider your life in general these days, would you say you are happy?”

Finally, a single question to assess for perceived discrimination was used as a preliminary screen of perceived discrimination to explore whether this construct is worth evaluating in more depth in a future full study. This question has been validated in women with postpartum depression and presents a simple, low burden method to evaluate for perceived discrimination [29]. The question: “Would you say that during the past 12 months someone treated you unfairly because of your gender, skin colour, the way you dress, your family origin, speech, religious beliefs or something else?” A simple yes/no response was recorded.

On completion of the interview, treatment and process data were collected from the participants’ folders. Data recorded included diagnosis, comorbidities, medication, medical and surgical cancer management.

### Procedure

The study adhered to the principles of the Declaration of Helsinki throughout with ethical approval granted by the University of Cape Town, Faculty of Health Sciences Human Research Ethics Committee (#766/2018). Subsequently institutional approval was obtained from GSH and the Radiation Oncology Department.

Potential study participants were identified from the weekly Breast Unit appointment list. These patients were contacted by telephone and invited to take part in a study of quality of life in breast cancer survivors during their visit to the Breast Unit the following week. Patients were reassured that participation in the study would in no way affect their ongoing treatment and no members of the data collection team were directly involved in patient care. On attending the GSH Breast Unit, the potential participants who had indicated an interest in participating were approached by the researchers and provided with the study information sheet in their language of choice and given the opportunity to have any questions answered. Participants who consented to take part then completed informed consent.

After agreeing to participate patients were escorted by the researchers into a private room for data collection. Participants were reassured that their place in the queue would not be lost while they were completing data collection. Data were collected by interview with the researchers reading each question from the battery of questionnaires to the participant in the language of their choice and recording their responses. Although the questionnaires were developed for self-administration, and were provided in participants’ language of choice, interviews were used to collect data to overcome the known low levels of reading and health literacy [30] and lack of familiarity with reading in an African home language (many patients are verbally fluent on their home language but not fluent in reading and writing) [31]. For the body chart diagram on the BPI, the participants were asked to shade areas of pain themselves. Participants were provided with a drink and snack while they completed the questionnaire. On completion of the questionnaire participants were asked if they would like to be invited to a presentation on completion of the study to learn about the study results. Contact details of participants who wished to attend a presentation were recorded. Participants who reported any pain, depression, anxiety or poor sleep were asked if they would like the researcher to inform the treating doctor. Participants not wanting the doctor to be informed were provided with contact information to relevant support or treatment options.

Raw data were kept in a locked cupboard in the Breast Unit and were collected weekly by the research team. Raw data were entered into an excel spread sheet by the research team and saved in password protected documents on a password protected computer.

### Data analysis

Prevalence of chronic pain was determined based on responses to the BPI. Participants were placed into one of two groups: Chronic Pain, or No Chronic Pain. Descriptive statistics were used to summarize the PSS, PMI and PIS of those with chronic pain in addition to summarizing the number of pain sites, type and distribution of pain.

The socio-demographic and health profiles of the participants were summarized for the entire sample and by group. Between group comparisons were performed using the Mann-Whitney U test or Spearman’s correlation coefficient calculated to determine differences in categorical variables. Statistical significance was accepted as p < 0.05.

## Results

**Fig. 1:**
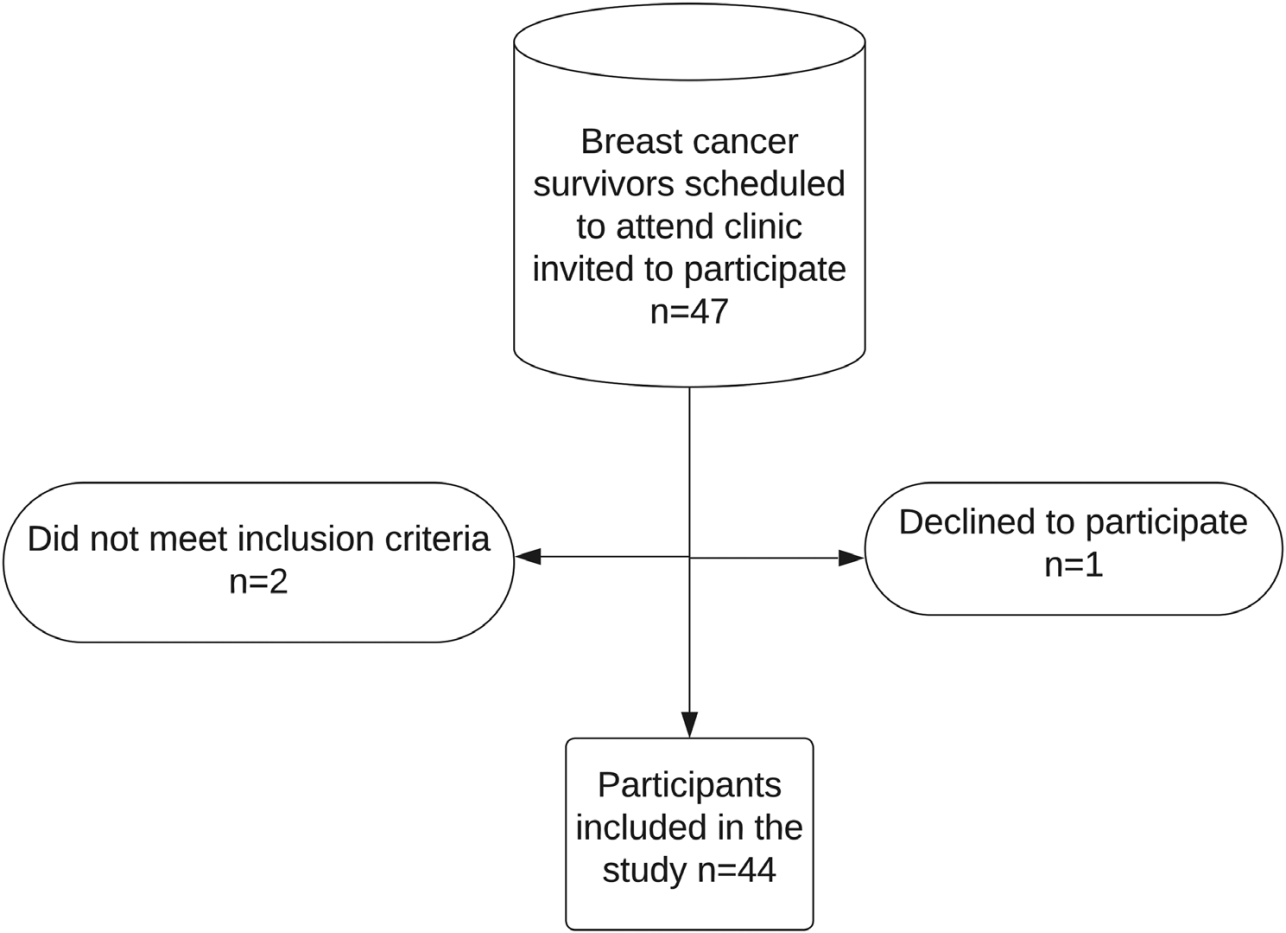
Participant recruitment

During the one month of the pilot study, 47 breast cancer survivors were scheduled to attend the Breast Unit and were invited telephonically to participate in the study. One patient declined to participate, and two patients were excluded on attending at the clinic as they did not meet inclusion criteria, leaving a sample of 44 patients (Fig. 1).

### Participant characteristics (n = 44)

The 44 women had a median age of 60.5 (IQR = 49.5–67.5). The majority were married, retired, had not completed school, chose to be interviewed in English (n = 42) and had low levels of health literacy (Table 1).

**Table 1:** Sociodemographic characteristics of the participants (n = 44)

Sociodemographic characteristic	Median (IQR)
Age	60.5 (49.5–67.5)
**Marital Status**	**Frequency (%)**
Married	20 (45)
Single	10 (23)
Widowed	8 (18)
Divorced	6 (14)
**Occupation**	
Retired	18 (41)
Employed	11 (25)
Unemployed	12(27)
Disabled (receiving a disability grant)	3 (7)
Highest level of education*	
Did not complete primary school	4 (9)
Completed primary school	1 (2)
Did not complete high school	26 (59)
Completed high school	4 (9)
Tertiary education	9 (21)
**Health literacy**	
Sufficient literacy	13 (30)
At risk	27 (61)
High risk	3 (7)
Extreme risk	1 (2)

^*In South Africa formal school is 12 years, primary school Grades 1–7, high school Grades 8–12^

### Health Profile (n = 44)

All the participants were breast cancer survivors. Eighteen (41%) of the women were receiving medication for the ongoing management of their breast cancer (4 on Arimidex; 14 Tamoxifen). For the management of their breast cancer, 24 (55%) of the women had been managed with surgery alone. Fourteen (32%) had been treated with surgery, chemotherapy and radiotherapy; 4 (9%) had received surgery and chemotherapy; 1(2%) had received surgery and radiotherapy and 1(2%) had received radiotherapy only. The majority of the women (28, 64%) self-reported that they had previously been diagnosed with other conditions, with 21 (48%) presenting with two or more co-morbidities (median 1, IQR = 0–2) (Table 2).

**Table 2:** Co-morbidities reported by the participants (n = 44)

Condition	Number (percentage)
Hypertension	20 (46)
Diabetes	7 (16)
Ischaemic heart disease	5 (11)
Hypercholesterolaemia	4 (9)
Arthritis	3 (7)
Cerebrovascular Accident	3 (7)
Asthma	2 (5)
Depressive disorder	2 (5)
Epilepsy	1 (2)
Hypothyroidism	1 (2)
Glaucoma	1 (2)
Cardiomyopathy	1 (2)
Hernia	1 (2)
Anxiety disorder	1 (2)
Arrhythmia	1 (2)
Hepatitis B	1 (2)
Cervical cancer	1 (2)

^Total is >100% as participants reported more than one co−morbidity^

The participants were receiving a range of medication for the management of their conditions (Table 3).

**Table 3:** Non-cancer related medications

Medication	Number (percentage)
Analgesics	19 (43)
Paracetamol/acetaminophen	8 (18)
Tramadol	8 (18)
Acetylsalicylic acid (aspirin)	2 (5)
Morphine	1 (2)
**Anti-hypertensives**	**26 (60)**
Hydrochlorothiazide	8 (18)
Atenolol	6 (14)
Amlodipine	4 (9)
Losartan	3 (7)
Enalapril	3 (7)
Furosemide	2 (5)
**Anti-diabetics**	**9 (20)**
Metformin	7 (16)
Insulin	1 (2)
Glimeperide	1 (2)
**Thyroid dysfunction**	**2 (4)**
Levothyroxine	1 (2)
Calcium carbonate	1 (2)
**Other medications**	
Warfarin	2 (5)
Simvastatin	4 (9)
Senna glycoside	2 (5)
Lansoprazole	2 (5)
Salbutamol inhaler	1 (2)
Sodium Valproate	1 (2)
Fluoxetine	1 (2)
Tamsulosin	1 (2)
Acrivastine	1 (2)
Vitamin D	1 (2)

### Pain

**Fig. 2:**
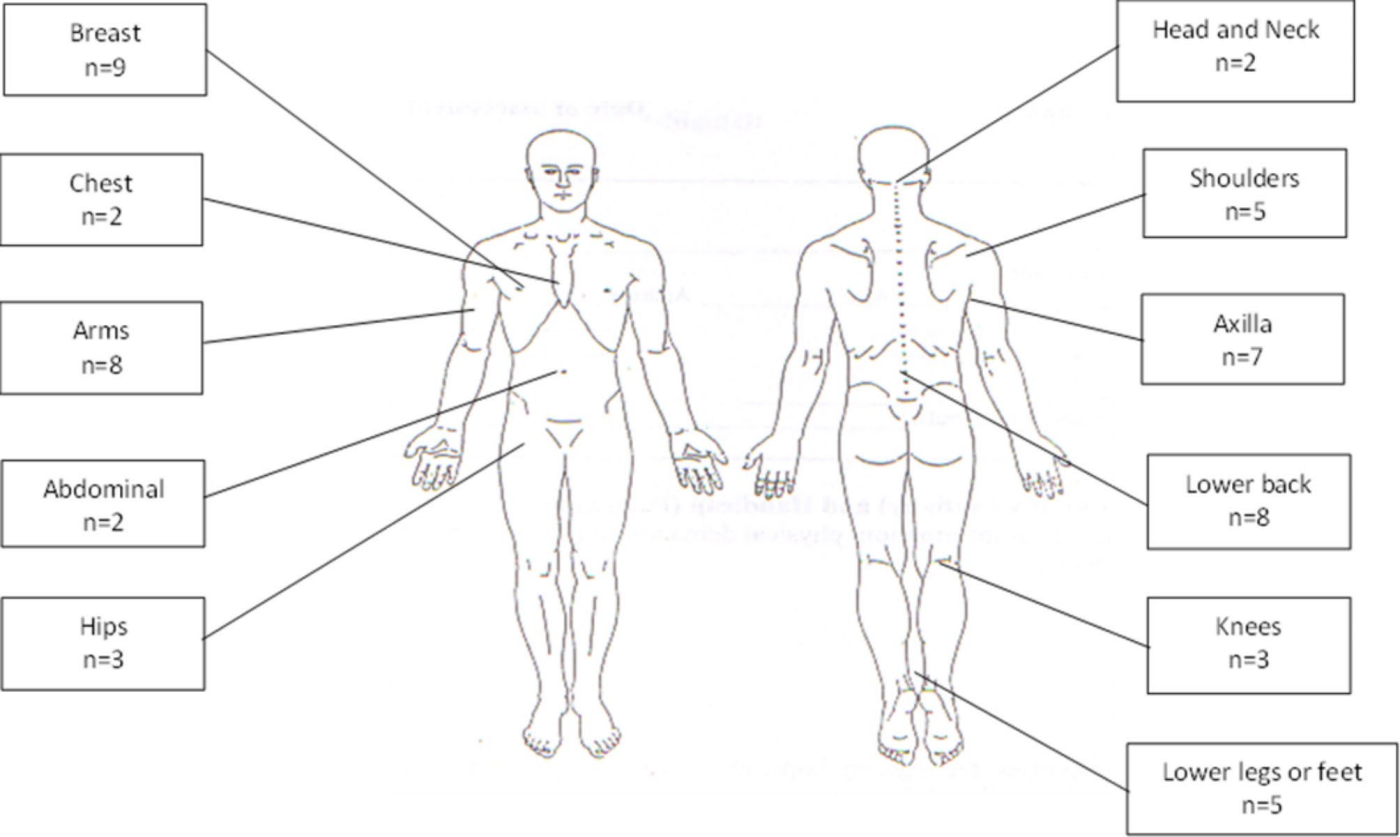
Sites of pain (n = 26)

There was a 59% prevalence of chronic pain (95%CI 44–72%), i.e. 26 of the women had experienced pain which they would not consider normal everyday types of pain on most days for three months or longer. The median pain severity score was 3.75 (IQR = 2.75-5) and the median pain interference with function score was 4 (IQR = 2.9–5.4) (Table 4). The women were experiencing pain in a median of 2 different body sites (IQR = 1–3) (Fig. 2). On the DN4, 17 (39%) of the women with pain were evaluated as presenting with neuropathic pain. However, only one of these women was being treated with medication appropriate for neuropathic pain (amitriptyline) with the others prescribed simple analgesics.

**Table 4:** Pain severity and pain interference scores on the Brief Pain Inventory (n = 26)

Category	Median (IQR)
Pain Severity Score	3.75 (2.75-5)
Worst pain	7 (5–9)
Least pain	2 (1–3)
Average pain	4 (2–5)
Pain right now	2 (0–4)
**Pain Interference with function**	**4 (2.57–5.4)**
General activity	5 (2–6)
Mood	5 (2–7)
Walking ability	2 (0–6)
Normal work	5 (2–6)
Relations with other people	2 (0–5)
Sleep	5 (2–7)
Enjoyment of life	4.5 (1–6)

^Scored on a 0–10 scale where 0=’no pain’ and 10=’worst possible pain’^.

^Pain Severity Score is calculated as the average of the worst, least, average and pain right now^.

^Pain interference with function is scored as the average of the 7 listed items^.

^Scores are interpreted as ‘mild pain’=0–3; ‘moderate pain’=4–6; ‘severe pain’=7–10^

The Pain Management Index (PMI) was calculated using the PSS and analgesic information. All of the women in pain were receiving analgesia and therefore had positive PMI scores. The majority of the women scored 2 on the PMI, suggesting that their pain was well managed.

### Sociodemographic characteristics and pain

The women with pain were significantly younger than the women without pain [53y (IQR = 48–64) vs. 66.5y (IQR = 56–73); (U = 121; p < 0.01)]. There were significant differences between groups in terms of occupation (Spearman r = 0.48; p < 0.01). None of the participants without pain were receiving a disability grant and only 2 (11%) were unemployed (Table 5).

**Table 5:** Differences in Sociodemographic Characteristics between those with pain and without pain

Sociodemographic characteristic	Women with pain n = 26	Women without pain n = 18	Statistical analysis
**Marital Status**	**Frequency (%)**	**Frequency (%)**	Spearman r = 0.19; p = 0.21
Married	11 (42)	9 (50)	
Single	10 (39)	0 (0)	
Widow	3 (12)	5 (28)	
Divorced	2 (8)	4 (22)	
**Income classification**			Spearman r = 0.25; p = 0.10
H0	9 (35)	8 (44)	
H1	15 (58)	4 (22)	
H2	2 (8)	5 (28)	
**Occupation**			Spearman r = 0.48; p < 0.01
Retired	6 (23)	12 (67)	
Employed	7 (27)	4 (22)	
Unemployed	10 (38)	2 (11)	
Disabled (receiving a disability grant)	3 (12)	0(0)	
Highest level of education*			Spearman r = 0.17; p = 0.26
Did not complete primary school	1 (4)	3 (17)	
Completed primary school	0 (0)	1 (6)	
Did not complete high school	18 (69)	8 (44)	
Completed high school	3 (12)	1 (6)	
Tertiary education	4 (15)	5 (28)	
**Health literacy**			Spearman r = 0.09; p = 0.53
Sufficient literacy	7 (27)	6 (33)	
At risk	17 (65)	10 (56)	
High risk	1 (4)	2 (11)	
Extreme risk	1 (4)	0 (0)	

^*indicates significance with p<0.05^

### Cancer management and pain

Eighteen of the women were on medication for the continued management of their breast cancer. There was no difference in medication between those with pain and without pain. There was also no difference in terms of cancer treatment between those with and without pain (Table 6).

**Table 6:** Cancer management in those with pain and without pain

	Women with pain n = 26	Women without pain n = 18	Statistical analysis
**Medication**	**Frequency (%)**	**Frequency (%)**	Spearman r = 0.19; p = 0.45
Arimidex	2 (8)	2 (11)	
Tamoxifen	10 (38)	4 (22)	
**Cancer management**			Spearman r = 0.22; p = 0.14
Surgery	17 (65)	7 (39)	
Surgery, chemotherapy, radiotherapy	8 (31)	6 (33)	
Surgery, radiotherapy	1 (4)	0 (0)	
Surgery, chemotherapy	0 (0)	4 (22)	
Radiotherapy	0 (0)	1 (6)	

### Health Related Quality of Life

On the EQ-5D health related quality of life instrument, the women with pain had significantly worse quality of life [70 (IQR = 50–80)] than the women without pain [85 (IQR = 70–90); U = 132.5; p = 0.01). For the individual health related quality of life domains, the women with pain scored significantly worse in the domains for “usual activities”; “pain/discomfort” and “anxiety/depression” Table 7.

**Table 7:** Problems in health-related quality of life domains in women with pain and women without pain

Health related quality of life domain	Women with pain n = 26	Women without pain n = 18	Statistical analysis
**Mobility**			Spearman r = 0.16; p = 0.29
No problems	18	15	
Some problems	8	3	
Unable to mobilise	0	0	
**Self-care**			Spearman r = 0.10; p = 0.51
No problems	23	17	
Some problems	3	1	
Extreme problems	0	0	
**Usual activities**			Spearman r = 0.37; p = 0.01*
No problems	12	15	
Some problems	14	3	
Extreme problems	0	0	
**Pain/Discomfort**			Spearman r = 0.45; p < 0.01*
No pain	3	11	
Moderate pain	22	7	
Extreme pain	1	0	
**Anxiety/Depression**			Spearman r = 0.54; p < 0.01*
None	6	14	
Moderate	19	4	
Extreme	1	0	

^*indicates significant difference between groups with p<0.01^

### Physical activity

On the IPAQ, there was no difference in the levels of physical activity in the women with pain compared to the women without pain (Spearman r = 0.27; p = 0.07) (Supplementary materials Table S1).

### Depression and anxiety

On the PHQ4, the participants with pain had significantly worse scores overall, as well as for the individual items assessing depression and anxiety (Supplementary materials Table S2). The women with pain had mild to moderate symptoms overall, with mild depression and mild to moderate anxiety.

### Pain catastrophizing

On the Pain Catastrophizing Scale, there was no difference in scores between those with pain vs. those without pain [3.5 (IQR = 0–8) vs. 0 (IQR = 0–12); U = 188.5; p = 0.28].

### Sleep, happiness and perceived discrimination

In response to the question asking whether participants had trouble falling or staying asleep or trouble sleeping too much, there was no difference between those with pain and those without pain (Spearman r = 0.22; p = 0.15). Overall, 23 of the women (52%) responded having trouble with sleep (16 with pain; 7 without pain).

In response to the question evaluating happiness, there was no difference between those with pain and those without pain (Spearman r = 0.23; p = 0.14). Overall, 41 of the women responded that they felt happy when considering their life in general (23 of those with pain and all 18 of those without pain).

In response to the question about perceived discrimination and whether they felt that during the past twelve months someone had treated them unfairly because of their gender, skin colour, the way they dress, family origin, speech or religious beliefs, there was no difference between those with pain and those without pain (Spearman r = 0.19; p = 0.20). Overall, 6 of the women responded feeling discriminated against in the past year, 5 of those with pain and 1 woman without pain.

## Discussion

We conducted a pilot study on the prevalence, characteristics and contributing factors to chronic pain in 44 breast cancer survivors. The majority of the women in our study were married (48%) and retired (43%). Most had not completed high school and had low levels of health literacy. The median age of these women was 60,5 (50–68), typical of women survivors of breast cancer internationally [32]. Similar to studies of breast cancer survivors elsewhere reporting high numbers of co-morbidities in this population, most of the women in our study had co-morbidities, with 21 (46%) presenting with two or more conditions [33]. In this setting, where the majority of patients present late in the course of their disease, management should include neoadjuvant chemotherapy or hormonal therapy to enable surgical resection. In this group of women, surgical management included mastectomy (18.39%), lumpectomy (4.9%) and wide local excision (1.2%). Subsequently, 18 (39%) of these women received chemotherapy and 16 (35%) were treated with radiotherapy.

The prevalence of chronic pain in this pilot study of breast cancer survivors was 59% (95% CI 44–72%), a significantly higher number than the 18.3% prevalence of chronic pain in adults reported in the South African demographic household survey [34]. While there is a wide variation in chronic pain prevalence estimates in breast cancer survivors, chronic pain prevalence appears to be consistently higher than in population samples [35]. This higher prevalence has been reported in breast cancer survivors from high-income countries such as the United States, (34.6% prevalence, near double the general population) [36], Denmark (42% prevalence) and Israel (74% prevalence of chronic pain) [37]; and from low- and middle-income countries including India (44% prevalence of chronic pain) [38] and Brazil ( [39].

In our cohort, there was a correlation between low levels of income and the prevalence of chronic pain. The social determinants of health, including the multiple aspects of poverty (low levels of income, low levels of education, poor access) have been demonstrated to contribute to the prevalence of chronic pain in a range of populations [40]. These findings support the need for attention to be given to the social determinants of health for breast cancer survivors to reduce burden on the individual and on the system. This will have to include other aspects beyond the health systems such as at the policy level and engagement with the distribution of resources and wealth. In a 2016 USA National Health Interview Survey, 20% of the population reported chronic pain with 8% of those having high impact chronic pain (chronic pain that frequently limits life or work activities) [40]. Chronic pain and high impact chronic pain were more prevalent in women; older adults; those unemployed and those living in poverty. The women receiving treatment at the Breast Unit are mostly from disadvantaged backgrounds and mostly from single income households. The majority were H1 classified which means that their treatment is subsidised by the government with patients paying a nominal fee of < ZAR1000 (US$53) for their treatment. To be classified as H1, patients must be earning less than ZAR70000 (US$3731) as single income or ZAR100000 (US$5331) family income per year. This income level is below the South African poverty line, clearly illustrated when the cost of a loaf of bread (US$0.75) or a litre of milk (US$1) are considered. These data suggest that the majority of these women struggle to make ends meet, thus making them more vulnerable or at high risk of developing chronic pain.

The women with pain were younger than those without, a finding previously reported in other studies of breast cancer survivors [41]. We hypothesise that younger breast cancer survivors may be more at risk for chronic pain due to the combined psychosocial challenges of self-stigma related to changes in body image, and the fear and anxiety of living with a potentially terminal condition from a young age.

The women with chronic pain had poorer health related quality of life than those without pain. They reported worse mobility in their usual activities, and pain or discomfort. This pushes us to strive to put measures in place to enhance the quality of life of cancer survivors [42]. A clear shift needs to be made by all members of the health team treating breast cancer beyond survival towards optimising quality of life. This extends beyond pain to mental health disorders. The women with chronic pain had more symptoms of depression and anxiety compared to the women without chronic pain. We did not explore whether the participants had received or pursued any non-pharmacological treatments for their pain, information which would be valuable to obtain in future studies. Depression and anxiety are recognised as risk factors for chronic pain [12], however, it may also be proposed that feeling depressed or anxious following treatment for breast cancer with ongoing pain illustrates a bidirectional relationship between depression/anxiety and chronic pain. Depression and anxiety may not be causative of the chronic pain but it certainly increases suffering and needs to be considered in treatment [43].

There were several strengths and weaknesses identified in this study which will inform a future larger study of chronic pain in breast cancer survivors. We were fortunate to be able to recruit enough candidates to provide adequate power for this pilot study. The selected measuring tools were easily understood and administered apart from the IPAQ. The IPAQ was particularly challenging to administer in this patient group with patients struggling to understand the wording of the questions and not relating to the questions. If levels of physical activity are to be evaluated in the larger study, an alternative measure such as the Yale Physical Activity Survey is recommended [44]. In addition, gender-specific body charts will be used as part of the BPI to enhance communication with participants.

The protocol for identifying potential participants and recruitment will be revised. The process of identifying eligible participants based on chart review alone was insufficient with interview checking of eligibility also required. The protocol required verbal telephonic consent to be obtained prior to approaching potential participants at the clinic. However, contacting patients telephonically prior to their clinic appointment was challenging with numerous potential participants not contactable thus reducing the potential sample. In-person recruitment on attendance at clinic would obviate this problem.

## Conclusion

The results of this pilot study suggest that chronic pain may be a significant burden in breast cancer survivors. A larger study in South African and African Breast Cancer survivors appears feasible and is indicated to explore this issue. We recommend that clinicians managing breast cancer survivors routinely assess for chronic pain at each clinical consultation using the Brief Pain Inventory to optimise the quality of life of these patients.

### Electronic supplementary material

Below is the link to the electronic supplementary material.

Supplementary Material 1

## Data Availability

The datasets generated and analyzed during the current study are not publicly available due to patient confidentiality and hospital policy but are available from the corresponding author on reasonable request.

## References

[1] Cumber SN, Nchanji KN, Tsoka-Gwegweni JM (2017). Breast cancer among women in sub-saharan Africa: prevalence and a situational analysis. South Afr J Gynaecol Oncol.

[2] Sung H, Ferlay J, Siegel RL, Laversanne M, Soerjomataram I, Jemal A, Bray F (2021). Global Cancer statistics 2020: GLOBOCAN estimates of incidence and Mortality Worldwide for 36 cancers in 185 countries. CA Cancer J Clin.

[3] Cancer IAfRo. : Latest world cancer statistics. *Global cancer burden rises to* 2013, 14.

[4] Organization WH. New global breast cancer initiative highlights renewed commitment to improve survival. In.: WHO; 2021.

[5] Reinertsen KV, Cvancarova M, Loge JH, Edvardsen H, Wist E, Fosså SD (2010). Predictors and course of chronic fatigue in long-term Breast cancer survivors. J Cancer Surviv.

[6] Variawa ML, Scribante J, Perrie H, Chetty S (2016). The prevalence of chronic postmastectomy pain syndrome in female Breast cancer survivors. South Afr J Anaesth Analgesia.

[7] Brinton LA, Figueroa JD, Awuah B, Yarney J, Wiafe S, Wood SN, Ansong D, Nyarko K, Wiafe-Addai B, Clegg-Lamptey JN (2014). Breast cancer in Sub-saharan Africa: opportunities for prevention. Breast Cancer Res Treat.

[8] Quinten C, Coens C, Mauer M, Comte S, Sprangers MA, Cleeland C, Osoba D, Bjordal K, Bottomley A (2009). Baseline quality of life as a prognostic indicator of survival: a meta-analysis of individual patient data from EORTC clinical trials. Lancet Oncol.

[9] Torrance N, Elliott AM, Lee AJ, Smith BH (2010). Severe chronic pain is associated with increased 10 year mortality. A cohort record linkage study. Eur J Pain.

[10] Muliira RS, Salas AS, O’Brien B (2017). Quality of Life among Female Cancer survivors in Africa: an Integrative Literature Review. Asia Pac J Oncol Nurs.

[11] Gärtner R, Jensen MB, Nielsen J, Ewertz M, Kroman N, Kehlet H (2009). Prevalence of and factors associated with persistent pain following Breast cancer Surgery. JAMA.

[12] Lahousse A, Roose E, Leysen L, Yilmaz ST, Mostaqim K, Reis F, Rheel E, Beckwée D, Nijs J (2022). Lifestyle and Pain following Cancer: state-of-the-art and future directions. J Clin Med.

[13] Poleshuck EL, Katz J, Andrus CH, Hogan LA, Jung BF, Kulick DI, Dworkin RH (2006). Risk factors for chronic pain following Breast cancer Surgery: a prospective study. J Pain.

[14] Schou Bredal I, Smeby NA, Ottesen S, Warncke T, Schlichting E (2014). Chronic pain in Breast cancer survivors: comparison of psychosocial, surgical, and medical characteristics between survivors with and without pain. J Pain Symptom Manage.

[15] Meijuan Y, Zhiyou P, Yuwen T, Ying F, Xinzhong C (2013). A retrospective study of postmastectomy pain syndrome: incidence, characteristics, risk factors, and influence on quality of life. ScientificWorldJournal.

[16] Adam AM. Sample size determination in Survey Research. J Sci Res Rep 2020:90–7.

[17] Jeppesen KM, Coyle JD, Miser WF (2009). Screening questions to predict limited health literacy: a cross-sectional study of patients with Diabetes Mellitus. Ann Fam Med.

[18] Parker R, Jelsma J, Stein DJ (2016). Pain in amaXhosa Women Living with HIV/AIDS: translation and validation of the brief Pain Inventory-Xhosa. J Pain Symptom Manag.

[19] Shraim MA, Sluka KA, Sterling M, Arendt-Nielsen L, Argoff C, Bagraith KS, Baron R, Brisby H, Carr DB, Chimenti RL (2022). Features and methods to discriminate between mechanism-based categories of pain experienced in the musculoskeletal system: a Delphi expert consensus study. Pain.

[20] Mulvey MR, Boland EG, Bouhassira D, Freynhagen R, Hardy J, Hjermstad MJ, Mercadante S, Pérez C, Bennett MI (2017). Neuropathic pain in cancer: systematic review, performance of screening tools and analysis of symptom profiles. Br J Anaesth.

[21] Jelsma J, Mkoka S, Amosun L, Nieuwveldt J (2004). The reliability and validity of the Xhosa version of the EQ-5D. Disabil Rehabil.

[22] Jelsma J, Ferguson G (2004). The determinants of self-reported health-related quality of life in a culturally and socially diverse South African community. Bull World Health Organ.

[23] Craig CL, Marshall AL, Sjöström M, Bauman AE, Booth ML, Ainsworth BE, Pratt M, Ekelund U, Yngve A, Sallis JF (2003). International physical activity questionnaire: 12-country reliability and validity. Med Sci Sports Exerc.

[24] Reyes-Gibby CC, Anderson KO, Morrow PK, Shete S, Hassan S (2012). Depressive symptoms and health-related quality of life in Breast cancer survivors. J Womens Health (Larchmt).

[25] Bhana A, Rathod SD, Selohilwe O, Kathree T, Petersen I (2015). The validity of the Patient Health Questionnaire for screening depression in chronic care patients in primary health care in South Africa. BMC Psychiatry.

[26] Morris LD, Grimmer-Somers KA, Louw QA, Sullivan MJ (2012). Cross-cultural adaptation and validation of the South African Pain Catastrophizing Scale (SA-PCS) among patients with fibromyalgia. Health Qual Life Outcomes.

[27] MacGregor KL, Funderburk JS, Pigeon W, Maisto SA (2012). Evaluation of the PHQ-9 item 3 as a screen for sleep disturbance in primary care. J Gen Intern Med.

[28] Calvo R, Arcaya M, Baum CF, Lowe SR, Waters MC (2015). Happily ever after? Pre-and-post Disaster determinants of Happiness among survivors of Hurricane Katrina. J Happiness Stud.

[29] Stepanikova I, Kukla L (2017). Is perceived discrimination in pregnancy prospectively linked to Postpartum Depression? Exploring the role of education. Matern Child Health J.

[30] Janse van Rensburg Z (2020). Levels of health literacy and English comprehension in patients presenting to South African primary healthcare facilities. Afr J Prim Health Care Fam Med.

[31] Khuluvhe M. Adult illiteracy in South Africa. In. Edited by Training DoHEa. Pretoria; 2021.

[32] Houssami N, Ciatto S, Martinelli F, Bonardi R, Duffy SW (2009). Early detection of second breast cancers improves prognosis in Breast cancer survivors. Ann Oncol.

[33] Leach CR, Weaver KE, Aziz NM, Alfano CM, Bellizzi KM, Kent EE, Forsythe LP, Rowland JH (2015). The complex health profile of long-term cancer survivors: prevalence and predictors of comorbid conditions. J Cancer Surviv.

[34] Kamerman PR, Bradshaw D, Laubscher R, Pillay-van Wyk V, Gray GE, Mitchell D, Chetty S (2020). Almost 1 in 5 South African adults have chronic pain: a prevalence study conducted in a large nationally representative sample. Pain.

[35] Peuckmann V, Ekholm O, Rasmussen NK, Groenvold M, Christiansen P, Møller S, Eriksen J, Sjøgren P (2009). Chronic pain and other sequelae in long-term Breast cancer survivors: nationwide survey in Denmark. Eur J Pain.

[36] Jiang C, Wang H, Wang Q, Luo Y, Sidlow R, Han X (2019). Prevalence of Chronic Pain and High-Impact Chronic Pain in Cancer survivors in the United States. JAMA Oncol.

[37] Hamood R, Hamood H, Merhasin I, Keinan-Boker L (2018). Chronic pain and other symptoms among Breast cancer survivors: prevalence, predictors, and effects on quality of life. Breast Cancer Res Treat.

[38] Asokan M, Asokan H, Mandal A, Cholakkal S, Babu B (2019). Prevalence of post-treatment chronic pain in Breast cancer patients and its effect on their quality of life. New Indian Journal of Surgery.

[39] Ferreira VT, Dibai-Filho AV, Kelly de Oliveira A, Gomes CA, Melo ES, Maria de Almeida A (2015). Assessing the impact of pain on the life of Breast cancer survivors using the brief Pain Inventory. J Phys Ther Sci.

[40] Dahlhamer J, Lucas J, Zelaya C, Nahin R, Mackey S, DeBar L, Kerns R, Von Korff M, Porter L, Helmick C (2018). Prevalence of Chronic Pain and High-Impact Chronic Pain among adults - United States, 2016. MMWR Morb Mortal Wkly Rep.

[41] Bao T, Seidman A, Li Q, Seluzicki C, Blinder V, Meghani SH, Farrar JT, Mao JJ (2018). Living with chronic pain: perceptions of Breast cancer survivors. Breast Cancer Res Treat.

[42] Ferrell BR, Grant MM, Funk BM, Otis-Green SA, Garcia NJ (1998). Quality of life in Breast cancer survivors: implications for developing support services. Oncol Nurs Forum.

[43] Gambassi G (2009). Pain and depression: the egg and the chicken story revisited. Arch Gerontol Geriatr.

[44] Semanik P, Lee J, Manheim L, Dipietro L, Dunlop D, Chang RW (2011). Relationship between accelerometer-based measures of physical activity and the Yale Physical Activity Survey in adults with arthritis. Arthritis Care Res (Hoboken).

