# Supplementary material for: Chronic pain in female breast cancer survivors - prevalence, characteristics and contributing factors: a cross-sectional pilot study
Source: BMC Womens Health. 2023 Nov 16;23:613. doi: 10.1186/s12905-023-02766-6 (PMC10655434; doi:10.1186/s12905-023-02766-6)
Supplement: Supplementary file 1 — Supplementary Material 1 [file 12905_2023_2766_MOESM1_ESM.docx]

**Supplementary Materials**

Table S1: Levels of physical activity reported on the IPAQ

| **Levels of physical activity** | **Women with pain**  **n=26** | **Women without pain**  **n=18** |
| --- | --- | --- |
| Low | 14 | 5 |
| Moderate | 7 | 9 |
| High | 5 | 4 |

Table S2: Difference in Depression and Anxiety in those with pain and without pain

| **Patient Health Questionnaire-4** | **Women with pain**  **n=26**  **Median (IQR)** | **Women without pain**  **n=18**  **Median (IQR)** | **Statistical analysis** |
| --- | --- | --- | --- |
| Total score | 5 (2-6) | 0 (0-5) | U=99.5; p<0.01* |
| Depression | 2 (1-3) | 0 (0-2) | U=101; p<0.01* |
| Anxiety | 3 (2-4) | 0 (0-2) | U=113.5; p<0.01* |
